# HDAC6 Regulates LPS-Tolerance in Astrocytes

**DOI:** 10.1371/journal.pone.0025804

**Published:** 2011-10-12

**Authors:** Eléonore Beurel

**Affiliations:** Department of Psychiatry and Behavioral Sciences, University of Miami, Miami, Florida, United States of America; Technische Universität München, Germany

## Abstract

Inflammatory tolerance is a crucial mechanism that limits inflammatory responses in order to avoid prolonged inflammation that may damage the host. Evidence that chronic inflammation contributes to the neuropathology of prevalent neurodegenerative and psychiatric diseases suggests that inflammatory tolerance mechanisms are often inadequate to control detrimental inflammation in the central nervous system. Thus, identifying mechanisms that regulate neuroinflammatory tolerance may reveal opportunities for bolstering tolerance to reduce chronic inflammation in these diseases. Examination of tolerance after repeated lipopolysaccharide (LPS) treatment of mouse primary astrocytes demonstrated that histone deacetylase (HDAC) activity promoted tolerance, opposite to the action of glycogen synthase kinase-3 (GSK3), which counteracts tolerance. HDAC6 in particular was found to be critical for tolerance induction, as its deacetylation of acetyl-tubulin was increased during LPS tolerance, this was enhanced by inhibition of GSK3, and the HDAC6 inhibitor tubacin completely blocked tolerance and the promotion of tolerance by inhibition of GSK3. These results reveal opposing interactions between HDAC6 and GSK3 in regulating tolerance, and indicate that shifting the balance between these two opposing forces on inflammatory tolerance can obliterate or enhance tolerance to LPS in astrocytes.

## Introduction

The inflammation response is a vital reaction to cell damage, disease, or infection, and includes intrinsic down-regulatory mechanisms that are crucial for limiting damage to the host [1]. These inhibitory processes include tolerance, the cumulative outcome of multiple down-regulating mechanisms that is characterized as reduced responses to repeated or extended exposure to an inflammatory stimulus, such as lipopolysaccharide (LPS) [2]. Tolerance mechanisms appear to be impaired relatively frequently in the central nervous system (CNS) because many neurological and psychiatric diseases are associated with excessive inflammation, exemplified by elevated levels of the pro-inflammatory cytokine interleukin-6 (IL-6) [3], [4], [5], [6], [7]. This dysregulated inhibitory control of neuroinflammation can promote disease susceptibility, exacerbate neuropathology, and hinder responses to therapeutic interventions. Thus, identification of tolerance mechanisms amenable to intervention may provide new strategies for controlling neuroinflammation to bolster therapeutic responses.

Neuroinflammation is mediated by the CNS resident immune cells, astrocytes and microglia [8], [9]. Although both cell types contribute to cytokine production in the CNS, the immune properties of astrocytes differ from those of microglia, including characteristics of inflammatory tolerance that contributes to limiting neuroinflammation [10]. Microglia, which share a common myeloid lineage with macrophages, display complete tolerance in the production of IL-6 in response to repeated stimulation with LPS [10], as do macrophages [11], so a second exposure to LPS fails to elicit IL-6 production by these cells. In contrast, astrocytes display a characteristic of semi-tolerance in response to repeated exposure to LPS, with IL-6 production diminished but not eliminated by pre-exposure to LPS [10]. In astrocytes, inflammatory tolerance is enhanced by inhibition of glycogen synthase kinase-3 (GSK3) [10], whereas in macrophages inflammatory tolerance is counteracted by inhibition of histone deacetylases (HDACs) [11]. These opposing regulatory influences of GSK3 and HDACs on inflammatory tolerance raised the possibility of interactions between GSK3 and HDACs in the modulation of tolerance. The class IIb HDAC, HDAC6 [12], has previously been shown to modify the regulation by GSK3 of β-catenin [13] and tau [14]. HDAC6 differs from most other HDACs in that HDAC6 is predominantly cytosolic and acetyl-tubulin is a major substrate [12]. These relationships prompted this study to test if HDAC6 is involved in regulating inflammatory tolerance, and the modulatory action of GSK3, on tolerance in astrocytes. The results demonstrate that HDAC6 is activated during LPS-tolerance, and inhibition of HDAC6 blocks LPS-induced tolerance of IL-6 production and the ability of GSK3 inhibitors to promote tolerance in astrocytes.

## Methods

### Ethics Statement

All mice were housed and treated in accordance with National Institutes of Health guidelines and procedures with mice were approved by the University of Alabama at Birmingham Institutional Animal Care and Use Committee (APN100508040).

### Reagents

Sources of chemicals were TDZD-8 (Calbiochem), sodium butyrate, valproic acid, trichostatin A (TSA), anacardic acid, 5′azacytidine, LiCl (Sigma), pargyline (Alexis), tubacin (a gift from Dr. Mazitschek) and CT99021 (University of Dundee, UK).

### Cell culture

Primary glia were prepared from the cerebral cortex of 1 day old C57Bl/6 mice or GSK3 knockin mice as described [15], and cultured in DMEM/F12 medium supplemented with 10% FBS, 0.3% glucose, 2 mM L-glutamine, 10 U/mL penicillin and 10 µg/mL streptomycin. For separation of astrocytes and microglia, after 10 days of culture the cells were shaken (30 h; 250 rpm), resulting in >99% pure astrocytes as determined by immunostaining with the astrocyte marker glial fibrillary acidic protein (GFAP). Astrocytes were cultured for up to four weeks for experiments, were trypsinized only twice, and were used at approximately 100% confluency in all experiments. Astrocytes were plated in different sized plate for experiments. After the first hour of shaking, the medium containing microglia cells was collected and microglia were cultured in the same medium as astrocytes with approximately 10^6^ cells per well in 24 well plates, and microglia were used in experiments within one week of isolation. RAW264.7 cells were cultured as described previously [16]. Bone marrow cells were isolated from the tibia of C57Bl/6 mice, and cultured for 6 days in the presence of M-CSF in RPMI 1640 medium supplemented with 10% FBS, 100 IU/mL penicillin, 100 µg/mL streptomycin, 1× nonessential amino acids, 1 µM sodium pyruvate, 2.5 µM β-mercaptoethanol and 2 mM L-glutamine, to obtain after 6 days of culture bone marrow-derived macrophages (BMM). Cells were left untreated (naive, 0) or stimulated with 100 ng/mL LPS for 24 h (to establish LPS-tolerance) in medium supplemented with 10% FBS, washed twice with warm medium, and given fresh media with all supplements (0/0) or with all supplements and 10 ng/mL LPS (0/LPS, LPS/LPS) for 1 h or 24 h. Where indicated, cells were treated with 10 µM CT99021, TDZD-8, 5′ azacytidine (AZA), anacardic acid (AA), or tubacin, 3 µM pargyline, 20 mM LiCl, 5 mM sodium butyrate (NaBu), 5 mM valproic acid (VPA), or 50 nM TSA.

### ELISA

IL-6 levels were measured by ELISA according to the manufacturer's instructions (eBioscience).

### Immunoblotting

Western blots were carried out as described previously [17] using antibodies to GSK3α/β (Millipore), GSK3β (BD Transduction), acetyl-tubulin, α-tubulin, HDAC6 and β-actin (Sigma).

### HDAC6 activity

HDAC6 activity was measured using a colorimetric HDAC6 assay kit (Abcam) according to the manufacturer's protocol. Cytosolic extracts were obtained using the nuclear kit extraction from Active Motif according to the manufacturer's protocol. tubacin-insensitive activity was subtracted from total activity to obtain the activity of HDAC6.

### Statistical analysis

Statistical significance between groups was evaluated by ANOVA with a post-hoc Dunnett's multiple comparison test where appropriate or by Student's t-test.

## Results

### HDAC inhibitors counteract LPS-induced semi-tolerance of IL-6 production in astrocytes

**Figure 1:**
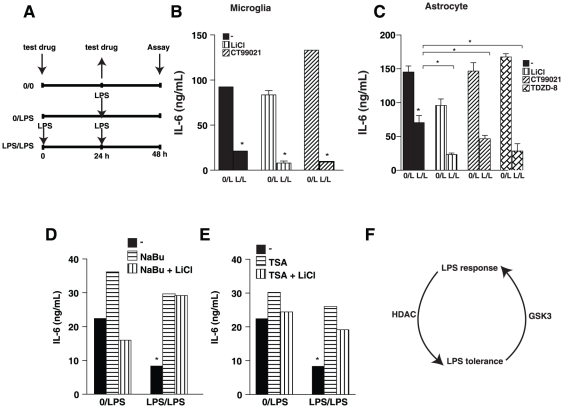
LPS-tolerance is blocked by HDAC inhibitors. *A*, Scheme of the LPS tolerance paradigm. Mouse primary microglia (*B*) or astrocytes (*C*) were pretreated where indicated with 100 ng/mL LPS (L) or no LPS (0) and the GSK3 inhibitors LiCl (20 mM), CT99021 (10 µM) or TDZD-8 (10 µM), for 24 h and restimulated with 10 ng/mL LPS for 24 h. Media were analyzed for IL-6 by ELISA. Data represent mean ± SEM, n = 3–6, *p<0.05 (ANOVA) in (*B*) compared with 0/L treatments, and in (*C*) compared with L/L in the absence of a GSK3 inhibitor. *D–E*, Mouse primary astrocytes were pretreated where indicated with 100 ng/mL LPS or no LPS (0) and the HDAC inhibitors 5 mM sodium butyrate (NaBu) (*D*), or 50 nM TSA (*E*), with or without 20 mM LiCl, for 24 h and restimulated with 10 ng/mL LPS for 24 h. Media were analyzed for IL-6 by ELISA. Data are representative of 3–4 experiments. *F*, Scheme of the opposing actions of GSK3 and HDAC in LPS tolerance.

Tolerance to LPS in macrophages was reported to be blocked by HDAC inhibitors [11]. Therefore, we tested if the HDAC inhibitors sodium butyrate, trichostatin A (TSA), and valproic acid blocked the development of semi-tolerance to LPS-stimulated IL-6 production and its facilitation by GSK3 inhibitors in astrocytes. As described previously [10], tolerance is determined by measuring the difference in IL-6 production by cells pre-exposed, or not, to LPS (Fig. 1A). Microglia preincubated with LPS produced little IL-6 upon restimulation with LPS, demonstrating full tolerance to LPS, which was not significantly modified by GSK3 inhibitors (Fig. 1B). Astrocytes preincubated with LPS produced ∼50% less IL-6 upon restimulation with LPS than did astrocytes not pre-exposed to LPS (Fig. 1C), demonstrating a phenotype of semi-tolerance. Astrocyte semi-tolerance to LPS was converted to more complete tolerance by co-treatment during the first exposure to LPS with GSK3 inhibitors, including lithium [18], [19], CT99021 [20] and TDZD-8 [21], as reported previously [10].

Opposite to the effects of GSK3 inhibitors, treatment with the HDAC inhibitors sodium butyrate (Fig. 1D) or TSA (Fig. 1E) during the first LPS stimulation of astrocytes completely blocked the induction of semi-tolerance in astrocytes. This demonstrates a requirement in astrocytes for active HDACs for the development of semi-tolerance to LPS in IL-6 production. Blockade of tolerance to LPS-induced IL-6 production by TSA in astrocytes matches the reported inhibition of tolerance by TSA in bone marrow-derived macrophages [11]. Examination of the effects of HDAC inhibitors on tolerance in primary microglia was not possible because the HDAC inhibitors induced rapid cell death in microglia, as reported previously [22].

To test whether inhibition of HDACs or GSK3 was dominant in regulating tolerance to LPS, astrocytes were treated with both types of inhibitors. This revealed that the promotion of tolerance induced by treatment with the GSK3 inhibitor lithium during the initial LPS stimulation was blocked by treatment with sodium butyrate (Fig. 1D) or TSA (Fig. 1E). Thus, active HDACs are required in order for inhibition of GSK3 to promote LPS-tolerance, as depicted in the scheme shown in Fig. 1F. In summary, these findings demonstrate that HDAC inhibitors counteract tolerance whereas GSK3 inhibitors promote tolerance [10], demonstrating the opposing actions of GSK3 (counteracting tolerance) and of HDACs (promotion of tolerance) in LPS-induced semi-tolerance in astrocytes.

**Figure 2:**
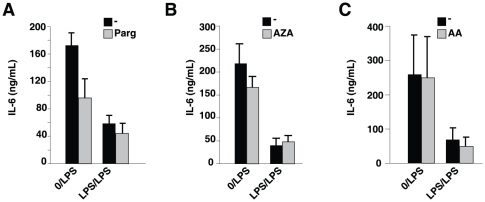
LPS-tolerance is not blocked by inhibitors of DNA methylase, H3 methylase or HATs in astrocytes. Astrocytes were pretreated where indicated with 100 ng/mL LPS or no LPS (0) and *A*, 3 µM pargyline (Parg), *B*, 10 µM 5′azacytidine (AZA) or *C*, 10 µM anacardic acid (AA) for 24 h and restimulated with 10 ng/mL LPS for 24 h. Supernatants were analyzed for IL-6 by ELISA (n = 3–4). Values represent mean ± SEM.

Unlike HDAC inhibitors, LPS-tolerance was unaffected by pretreatment with pargyline, an inhibitor of H3 demethylase LSD1 (Fig. 2A), 5'azacytidine, a DNA methylase inhibitor (Fig. 2B), or the HAT inhibitor anacardic acid (Fig. 2C), emphasizing the selective involvement of HDACs in the astrocytic LPS-semi-tolerance.

### HDAC6 promotes LPS-tolerance

**Figure 3:**
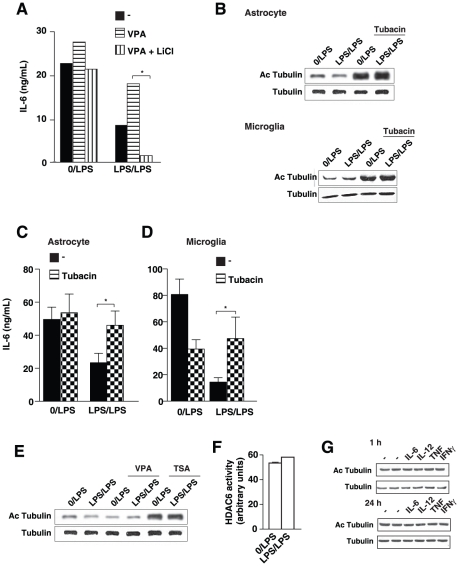
HDAC6 inhibition counteracts tolerance. *A*, Mouse primary astrocytes were pretreated where indicated with 100 ng/mL LPS or no LPS (0) and the HDAC inhibitor 5 mM valproic acid (VPA), with or without 20 mM LiCl, for 24 h and restimulated with 10 ng/mL LPS for 24 h. Media were analyzed for IL-6 by ELISA. Data are representative of 3–4 experiments, *p<0.05, ANOVA. *B*, Mouse primary astrocytes (*top panel*) or microglia (*bottom panel*) were pretreated where indicated with 100 ng/mL LPS or no LPS (0) with or without the HDAC6 inhibitor tubacin (10 µM) for 24 h and restimulated with 10 ng/mL LPS for 1 h. Cell extracts were immunoblotted for acetyl-α-tubulin (Ac Tubulin), and re-blotted for total α-tubulin (n = 3). Mouse primary astrocytes (*C*) or microglia (*D*) were pretreated where indicated with 100 ng/mL LPS or no LPS (0) and the HDAC6 inhibitor tubacin (10 µM) for 24 h and restimulated with 10 ng/mL LPS for 24 h. Media were analyzed for IL-6 by ELISA. Data represent mean ± SEM, n = 6, *p<0.05, ANOVA. *E*, Mouse primary astrocytes were pretreated where indicated with 100 ng/mL LPS or no LPS (0) and the HDAC inhibitors 5 mM valproic acid (VPA) or 50 nM TSA for 24 h and restimulated with 10 ng/mL LPS for 1 h. Cell extracts from astrocytes were immunoblotted for acetyl-α-tubulin, and re-blotted for total α-tubulin (n = 4). *F*, Mouse primary astrocytes were pretreated with 100 ng/mL LPS or no LPS (0) for 24 h and restimulated with 10 ng/mL LPS for 1 h. HDAC6 activity was measured in cytosolic extracts. The histogram represents mean ± SEM of the tubacin-inhibited HDAC activity. *G*, Mouse primary astrocytes were treated with 10 ng/mL of either IL-6, IL-12, TNFα, or IFNγ for 1 or 24 h. Cell extracts from astrocytes were immunoblotted for acetyl-α-tubulin, and re-blotted for total α-tubulin.

In contrast to sodium butyrate and TSA, treatment of astrocytes with the HDAC inhibitor valproic acid did not block the promotion by lithium of LPS-tolerance in IL-6 production (Fig. 3A). Valproic acid inhibits the same HDACs as sodium butyrate and TSA except for HDAC6 and HDAC10, which are not inhibited by valproic acid [23]. The capacity of lithium to promote LPS-tolerance in the presence of valproic acid but not with sodium butyrate or TSA suggested that this action of lithium may involve HDAC6 or HDAC10, indicating that these may be a target of GSK3 to counteract LPS-tolerance.

**Figure 4:**
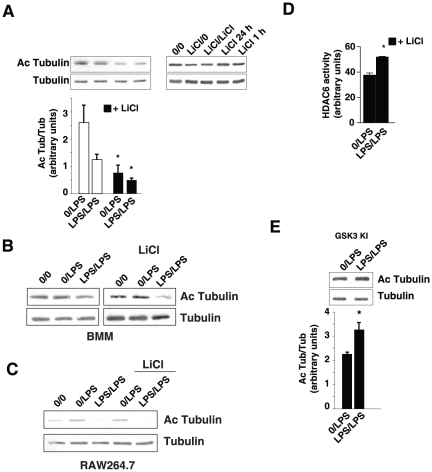
Lithium increases HDAC6 activity to promote tolerance. *A*, Mouse primary astrocytes were pretreated where indicated with 100 ng/mL LPS or no LPS (0) and the GSK3 inhibitor LiCl (20 mM) for 24 h (*left panel*) or the indicated time (*right panel*) and restimulated with 10 ng/mL LPS for 1 h (*left panel*) or LiCl (20 mM) for 24 h or the indicated time (*right panel*). Cell extracts were immunoblotted for acetyl-α-tubulin, and re-blotted for total α-tubulin. The ratio of acetylated-tubulin to total α-tubulin was quantitated. Histograms represent mean ± SEM, n = 5, *p<0.05, ANOVA compared to results in cells not treated with lithium. Mouse primary BMM (*B*) or RAW267.1 cells (*C*) were pretreated where indicated with 100 ng/mL LPS or no LPS (0) and the GSK3 inhibitor LiCl (20 mM) for 24 h and restimulated with 10 ng/mL LPS for 1 h. Cell extracts were immunoblotted for acetyl-α-tubulin, and re-blotted for total α-tubulin (n = 3). *D*, Mouse primary astrocytes were pretreated where indicated with 100 ng/mL LPS or no LPS (0) and the GSK3 inhibitor LiCl (20 mM) for 24 h and restimulated with 10 ng/mL LPS for 1 h. HDAC6 activity was measured in cytosolic extracts. The histogram represents mean ± SEM of the tubacin-inhibited HDAC activity, n = 3, *p<0.05, ANOVA. *E*, Mouse primary astrocytes isolated from GSK3 knockin mice were pretreated where indicated with 100 ng/mL LPS or no LPS (0) for 24 h and restimulated with 10 ng/mL LPS for 1 h. Cell extracts were immunoblotted for acetyl-α-tubulin, and re-blotted for total α-tubulin. The ratio of acetylated-tubulin to total tubulin was quantitated (mean ± SEM, n = 4, *p<0.05, ANOVA).

To specifically examine the role of HDAC6 in regulating tolerance, we tested if inhibiting HDAC6 with tubacin, a small molecule selective inhibitor of HDAC6 [24], was sufficient to promote LPS-tolerance. Acetylated-tubulin is a substrate of HDAC6, so increased acetyl-tubulin is a marker of HDAC6 inhibition [24], [25]. Treatment with tubacin markedly increased acetylated-tubulin (Fig. 3B) and completely blocked LPS-tolerance of IL-6 production in astrocytes (Fig. 3C), demonstrating that HDAC6 is required for LPS-tolerance in astrocytes. Tubacin also increased acetylated-tubulin in microglia (Fig. 3B) and appeared to counteract LPS-tolerance in microglia (Fig. 3D). However, in microglia pretreatment with tubacin alone induced a significant reduction of IL-6 production after one stimulation with LPS, suggesting an important role of HDAC6 in the microglial response to LPS, which limits conclusions about the role of HDAC6 in inflammatory tolerance in microglia. That HDAC6 is particularly important in the induction of LPS-induced semi-tolerance in astrocytes was further indicated by the finding that induction of LPS-tolerance was associated with a 50% decrease in acetyl-tubulin in LPS-tolerant astrocytes compared with a single exposure to LPS (Figs. 3E and 4A), indicative of activation of the HDAC6-mediated deacetylation of acetyl-tubulin during tolerance. However, this was not due to a generalized increase in HDAC6 activity, as HDAC6 activity in whole cell lysates (not shown) and in cytosolic fractions (Fig. 3F) was equivalent in astrocytes treated with LPS once or for two sequential periods. Treatment with TSA, but not valproic acid, also blocked the decrease in acetyl-tubulin caused by the LPS/LPS treatment (Fig. 3E), matching their differential modulatory effects on semi-tolerance in IL-6 production and the inhibition of HDAC6 by TSA but not by valproic acid [23].

LPS-stimulated TLR4 results in changes in the production of multiple cytokines that could contribute to changes in HDAC6. To begin to test if inflammatory cytokines may mediate the modulation of HDAC6 following LPS treatment, we tested in primary astrocytes if four cytokines, IL-6, IL-12, TNFα, and IFNγ altered the activity of HDAC6 as indicated by changes in acetyl-tubulin. Treatment of primary astrocytes for 1 or 24 hr with 10 ng/mL of either IL-6, IL-12, TNFα, or IFNγ did not alter acetylated tubulin (Fig. 3G), indicating that signaling mechanisms other than these cytokines mediate the change in HDAC6 elicited by LPS treatment.

### GSK3 counteracts tolerance through inhibition of HDAC6

Inhibition of GSK3 with lithium, which promotes tolerance, decreased acetyl-tubulin levels in conjunction with promoting LPS-induced tolerance, whereas treatment with lithium alone in the absence of LPS did not alter acetyl-tubulin (Fig. 4A). GSK3 inhibition in conjunction with LPS/LPS treatments also decreased acetyl-tubulin in primary bone marrow-derived macrophages (Fig. 4B) and RAW264.7 cells (Fig. 4C), demonstrating this is not a cell type-dependent action. Examination of HDAC6 activity in cytosolic extracts (Fig. 4D) also demonstrated a significant increase in HDAC6 activity in the presence of lithium during LPS-induced tolerance, indicating that GSK3 decreases HDAC6 activity during LPS tolerance.

Oppositely to examining the effects of GSK3 inhibitors on semi-tolerance in astrocytes, the effects of increased GSK3 activity were assessed by using astrocytes prepared from GSK3 knockin mice. This tactic was used instead of overexpressing GSK3 because previous studies have shown that overexpression of GSK3β in astrocytes causes apoptosis [26]. The two isoforms of GSK3 are predominantly regulated by inhibitory phosphorylation on serine-21-GSK3α and serine-9-GSK3β [27]. Examination of the effects of constitutively maximal GSK3 activity can be studied using homozygous GSK3α^21A/21A^/β^9A/9A^ knockin mice, where the regulatory serines of both GSK3 isoforms are mutated to alanines [28], which maintain GSK3 maximally active, but within the physiological range. In astrocytes from GSK3 knockin mice, there was no induction of LPS semi-tolerance [10]. Furthermore, there was no decrease in acetyl-tubulin after LPS/LPS treatment, but rather an increase, in astrocytes from GSK3 knockin mice (Fig. 4E). Thus, the blockade of LPS-induced semi-tolerance in astrocytes expressing fully active GSK3 was associated with a block in LPS-induced HDAC6 activation. These results demonstrate that LPS-tolerance requires inhibition of GSK3 to reduce GSK3-dependent inhibition of HDAC6.

### GSK3 associates with HDAC6

**Figure 5:**
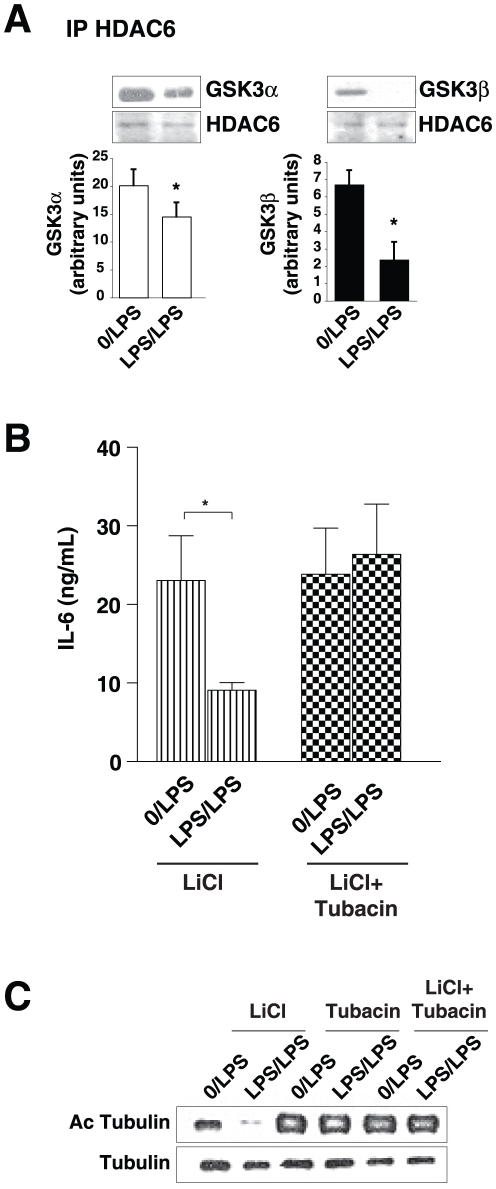
HDAC6 inhibition counteracts promotion of tolerance by lithium. *A*, Astrocytes were pretreated where indicated with 100 ng/mL LPS or no LPS (0) for 24 h and restimulated with 10 ng/mL LPS for 1 h. HDAC6 was immunoprecipitated from cell lysates, immunoprecipitants were immunoblotted for GSK3α, GSK3β, and HDAC6, and GSK3 co-immunoprecipitation was quantified (n = 3, mean ± SEM, *p<0.05, Student's t-test). *B*, Astrocytes were pretreated where indicated with 100 ng/mL LPS or no LPS (0) and the GSK3 inhibitor lithium (20 mM) with or without the HDAC6 inhibitor tubacin (10 µM) for 24 h and restimulated with 10 ng/mL LPS for 24 h. Media were analyzed for IL-6 by ELISA. Data represent mean ± SEM, *p<0.05, ANOVA. *C*, Mouse primary astrocytes were pretreated where indicated with 100 ng/mL LPS or no LPS (0) and the GSK3 inhibitor lithium (20 mM) with or without the HDAC6 inhibitor tubacin (10 µM) for 24 h and restimulated with 10 ng/mL LPS for 1 h. Cell extracts were immunoblotted for acetyl-α-tubulin, and re-blotted for total α-tubulin (n = 3).

To test if HDAC6 inhibition by GSK3 may be a direct effect, co-immunoprecipitation was used to test if the proteins were associated. Both GSK3α and GSK3β co-immunoprecipitated with HDAC6 (Fig. 5A). Furthermore, the association of HDAC6 with GSK3 was significantly decreased in tolerant LPS/LPS-stimulated astrocytes, demonstrating that tolerance is associated with dissociation of inhibitory GSK3 from HDAC6 to permit HDAC6 to promote tolerance.

To analyze if GSK3 by inhibiting HDAC6 modulates IL-6 production, we examined the effects of tubacin on lithium promotion of LPS-tolerance in IL-6 production. The promotion by lithium of LPS-tolerance in IL-6 production was abolished in the presence of the HDAC6 inhibitor, tubacin. To confirm that tubacin blocks the effects of lithium of HDAC6 activity, we also examined acetylated-tubulin levels and found that tubacin prevented the reduction by lithium of tubulin acetylation. Taken together, these results demonstrated that HDAC6 activity is increased by LPS-tolerance and this is counteracted by active GSK3.

## Discussion

Inflammation in the CNS can have particularly detrimental consequences if it damages neurons, which cannot be replaced. Since markers of excessive neuroinflammation have been identified in association with many neurodegenerative and psychiatric diseases, it is important to devise interventions that can control neuroinflammation [4], [5], [6]. One potential method to protect the CNS from irreversible damage induced by inflammation is to bolster endogenous mechanisms of inflammatory tolerance, which is characterized by the dampening of inflammatory responses to repeated inflammatory stimuli. Although microglia appear to exhibit full inflammatory tolerance to LPS similar to the closely related peripheral macrophages, astrocytes exhibit less complete tolerance, providing an opportunity to identify interventions capable of bolstering the development of tolerance. However, little is known about inflammatory tolerance mechanisms in the CNS, in contrast to the periphery where LPS-tolerance has been well-characterized. Tolerance mechanisms identified in the periphery include the induction of anti-inflammatory cytokines (e.g., IL-10, TGFβ) to counteract the inflammatory response [29], [30], [31], [32], [33] and down-regulation of the LPS-responsive TLR4 signaling pathway [34], including internalization of TLR4 from the cell surface [35], reduced activation of the inflammatory cytokine-inducing transcription factor NF-κB [36], [37], and chromatin modifications leading to the extinction of the pro-inflammatory cytokine gene expression [11]. Following induction of semi-tolerance to LPS in astrocytes, we did not observe an increase of anti-inflammatory cytokine production [10] or a reduction in the surface expression of TLR4, but we found decreases in the activation of both NF-κB and STAT3 [[10]; and data not shown]. Thus, mechanisms regulating tolerance in astrocytes appear to differ from those exhibited by peripheral immune cells.

This study found that the activity of the class IIb histone deacetylase, HDAC6, is important for promoting LPS-tolerance of IL-6 production in astrocytes, and that one mechanism by which GSK3 counteracts LPS-tolerance is by inhibition of HDAC6. These mechanisms indicate that inflammatory conditions in the CNS associated with inhibition of HDAC6 or activation of GSK3 can impede the development of LPS-tolerance, which may heighten deleterious pathological outcomes to inflammation. Identification of a role for HDAC6 in promoting inflammatory tolerance in astrocytes does not affect the likelihood that other HDACs are also capable of modulating the development of inflammatory tolerance in astrocytes as occurs in peripheral immune cells [11], [38]. This is evident from the capacity of valproic acid, which inhibits HDACs other than HDAC6 and HDAC10, to partially impede the development of semi-tolerance in astrocytes (Fig. 3). However, identification of a regulatory role for HDAC6 in tolerance fits well with previous evidence that HDAC6 has a suppressive effect on MyD88-dependent signaling by TLRs [39]. Thus, the present results extend the known functions of HDAC6 in immune cells to also promote LPS-tolerance in astrocytes. Although total cellular HDAC6 activity was not increased during LPS tolerance, we observed a significant reduction of the acetylation of α-tubulin during LPS-tolerance, indicative of increased HDAC6 action in a subcellular compartment associated with tubulin, and greatly increased tubulin acetylation after treatment with the HDAC6 inhibitor tubacin, which also blocked LPS-tolerance. These findings are consistent with a previous study showing that HDAC6 deacetylase activity links the tubulin cytoskeleton with immune synapse organization, whereas overexpression of HDAC6 greatly impaired the production of IL-2 [40]. The blockade of LPS-tolerance in IL-6 production was also observed in microglia treated with tubacin, suggesting a more generalized role of HDAC6 during LPS tolerance of IL-6 production. However, the precise mechanism by which HDAC6 promotes tolerance remains to be identified, which may involve its regulation of acetyl-tubulin or other acetylated proteins. Since HDAC6 has multiple cellular substrates, it is unknown which of these mediates its promotion of tolerance, but a potential mechanism is that regulation of acetyl-tubulin by HDAC6 alters intracellular transport and signaling mechanisms, including disruption of cytokine release.

The regulation of LPS tolerance by HDAC6 was also found to be linked to the previously identified role of GSK3 in counteracting LPS-tolerance [10]. Upon inhibition of HDAC6 with tubacin, as well as with TSA and sodium butyrate treatments, there was a complete block of the promotion of tolerance by lithium, which we previously demonstrated was due to its inhibition of GSK3 [10], whereas inhibition of GSK3 promoted HDAC6 activity and LPS-tolerance. Thus, these findings reveal opposing actions of HDAC6 and GSK3 in regulating LPS-tolerance, as HDAC6 promotes tolerance whereas GSK3 counteracts tolerance. A similar opposing action has been described in other systems, where HDAC6 blocks phosphorylation of β-catenin, whereas GSK3 phosphorylates β-catenin to promote its degradation [13]. Moreover, an indirect action of HDAC6 on GSK3 activity may be mediated by HDAC6 binding to the catalytic subunit of protein phosphatase-1 (PP1) [41] which promotes PP1 activity, which would lead ultimately to activation of GSK3 [42] to impede LPS tolerance. In contrast, another study found that HDAC6 is required for GSK3 phosphorylation of the microtubule-associated protein tau [14]. These findings suggest multiple regulatory interactions between HDAC6 and GSK3, including potential direct interactions that is indicated by their co-immunoprecipitation (Fig. 5A), that have context-specific functional outcomes on the actions of HDAC6 and GSK3.

In summary, this study reports a new mechanism of regulation of LPS-tolerance in astrocytes by the opposing actions of GSK3 and HDAC6. Thus, GSK3 inhibitors can promote LPS-tolerance, whereas inhibition of HDAC6 counteracts LPS-tolerance. Therefore, treatments that shift the balance between these two opposing forces on inflammatory tolerance can obliterate or enhance tolerance to LPS in astrocytes.
